# Food allergy and anaphylaxis – 2056. Clinical cross-reactivity of major food allergens among children

**DOI:** 10.1186/1939-4551-6-S1-P139

**Published:** 2013-04-23

**Authors:** Miho Hasegawa, Takatsugu Komata, Kiyotake Ogura, Katsuhito Iikura, Sakura Sato, Morimitsu Tomikawa, Akinori Shukuya, Motohiro Ebisawa

**Affiliations:** 1 Clinical Research Center for Allergy and Rheumatology, Sagamihara National Hospital, Japan; 2 Natinal Sagamihara Hospital, Japan; 3 Sagamihara National Hospital, Japan; 4 Department of Pediatrics, Natinal Sagamihara Hospital, Sagamihara, Japan; 5 Department of Allergy, Sagamihara National Hospital, Clinical Research Center, Japan

## Background

There are some reports on cross-reactivity among food allergens based on serological tests. On the other hand, actual frequency of cross-reactivity among food allergens based on clinical symptoms or results of oral food challenge (OFC) has not been reported.

## Methods

We sought to clarify information of elimination diets related to clinical cross-reactivity of childhood major food allergens (hen’s egg, cow’s milk, wheat, soybean, and peanuts). We reviewed clinical records of patients who had visited to our department from January to December in 2010. One thousand eight hundreds twenty-two patients (1207 males and 615 females) were recruited to this study.

## Results

Patients’ profiles were as follows; average age 5y8m+/-3y8m (mean+/-SD); number of eliminated foods 2.1+/-1.4 items per patient. Patients had been diagnosed mostly based on definitive clinical symptoms or results of OFC, but some patients had avoided foods based on elevated antigen-specific IgE tests. In 1226 patients with hen’s egg allergy, only 2 patients (0.2%) had avoided chicken, 44 (3.6%) salmon roe, and 16 (0.1%) other kind of fish eggs. In 771 cow’s milk allergy patients, 3 patients (0.4%) had avoided beef. In 392 wheat allergy patients, 5 patients (1.2%) had removed other grains such as barley and rye. In 72 soybean allergy patients, 23 patients (31.9%) had removed peanuts, on the other hand, in 445 patients avoiding peanuts, 24 (5.4%) had avoided soybean. Moreover 81 of 445 (18.2%) patients avoiding peanuts had eliminated tree nuts. Seventy-two patients with soy bean allergy had not avoided any other legumes.

## Conclusions

Based on definitive clinical symptoms or results of OFC, we could reveal that frequency of elimination diets associated with cross-reactivity of the major food allergens was much lower than that reported by serological tests.

